# Supplementary material for: Are carbon emissions trading and green financial instruments synergistic? -Comprehensive quantitative research based on content analysis
Source: PLoS One. 2024 Mar 7;19(3):e0298601. doi: 10.1371/journal.pone.0298601 (PMC10919601; doi:10.1371/journal.pone.0298601)
Supplement: S1 Data — (ZIP) [file pone.0298601.s001.zip › Data/地级市Z_F工作报告.zip!/║■─╧/║Γ╤⌠╩╨/└┤╫╘╣1⁄2╓┌║┼í╛┬φ┐╦╩2╛▌═°í┐_2017.docx]

各位代表：

现在，我代表市人民政府，向大会作政府工作报告，请予审议，并请各位政协委员和其他列席人员提出意见。

一、本届政府工作回顾

市十四届人大一次会议以来的四年，是我市发展进程中极具挑战、极不平凡的四年。在省委、省政府及市委的坚强领导下，市政府团结、带领和依靠全市人民，积极适应经济发展新常态，有效应对经济下行压力，坚持以发展提振信心、以发展重塑形象，经济社会发展呈现稳中有进的良好态势，谱写了加快全面建成小康衡阳的新篇章。

四年来，我们主要做了以下工作：

一是积极适应新常态，综合实力迈上新台阶。从经济总量看：地区生产总值2013年突破2000亿元，2016年达2820亿元（预计数，下同），年均增长9.1%；一般公共预算收入2013年突破200亿元，2016年达277.96亿元，年均增长10.7%；社会消费品零售总额2013年突破700亿元，2016年达1127亿元，年均增长12.6%，主要经济指标增长稳定性增强，综合实力稳居全省第一方阵。全市全面小康实现程度预计达91%，蒸湘区获评全省首批全面小康达标县（市），珠晖区获评全省全面小康推进工作十快进县。从发展支撑看：项目个数和投资规模连创新高，累计实施重点项目1763个、完成投资3291亿元，投资对经济增长的贡献率保持在55%以上。累计争取国省资金850亿元。深入开展精准招商，20家世界500强企业和18家央企来衡发展，富士康工业新城、五矿金铜、中兴网信等一大批产业项目形成新的经济增长点。从创新驱动看：大众创业、万众创新扎实推进，新技术新业态新模式不断涌现，全市市场主体达20.86万户。多层次资本市场加快发展，新增上市企业13家，湖南机油泵成功在A股上市。创新创业园区“135”工程建设推进有力，累计建成标准厂房369.8万平方米。智能制造和“互联网+”行动扎实推进，高新技术产业增加值年均增长21.7%。发明专利申请量和授权量年均分别增长47.4%、30.7%，衡山获评全国知识产权强县工程试点县，耒阳获评国家知识产权试点城市。

二是加快转型升级步伐，产业发展开拓新局面。三次产业结构优化为15.1∶43.9∶41.0，三产占比较2012年提升6个百分点。现代农业加速转型。粮食年产量稳定在330万吨以上，收购量稳居全省第一，获评全国粮食生产先进市。油茶、有机茶、酥脆枣等特色产业不断发展壮大，获评全国油茶产业发展示范市。市级以上农业产业化龙头企业达338家，农产品加工业总产值年均增长25%。在全省率先建成农业农村信息化示范基地。完成水利工程建设32.86万处，衡邵干旱走廊综合治理成效明显。新型工业加速推进。克服清理历史虚增等影响，工业保持平稳健康发展，规模工业增加值年均增长8.7%。天雁机械、湖南机油泵、南岳电控等20多家企业在全国细分行业处于领先地位。项目环保、能耗准入门槛明显提高，六大高耗能行业增加值占规模工业增加值比重较2012年下降4.6个百分点。市直“两区一园”完成调规扩区，衡山科学城、白沙绿岛军民融合产业园启动建设，全市园区新增建成区面积51.45平方公里；获批国家级高新技术产业开发区、国家循环化改造示范试点园区、国家现代农业科技示范园区；省级以上园区规模工业增加值占全市规模工业增加值比重达62.6%，较2012年提升31.8个百分点。现代服务业加速壮大。国家服务业综合改革试点工作顺利完成，服务业对经济增长的贡献率达56.5%，较2012年提高15.8个百分点。红星美凯龙、中建衡阳中心等城市综合体形成消费新热点，丽波酒店、崇盛国际中心加快建设，万达、碧桂园、恒大等知名企业纷纷抢滩登陆，华耀城、衡缘物流中心等物流枢纽项目建设取得突破性进展。电子商务来势喜人，电商年交易额突破100亿元，成为全国首批信息消费试点市。金融事业蓬勃发展，全市金融机构本外币各项存、贷款余额分别达3300亿元、1423亿元，年均分别增长16.5%、19.5%。旅游经济持续升温，接待国内外游客、实现旅游总收入年均分别增长12.6%、17.8%，新增3A级以上景区9个，南岳区获批“国家全域旅游示范区”创建单位，耒阳被评为全国休闲农业与乡村旅游示范县。

三是全面深化改革开放，内生动能激发新活力。供给侧结构性改革推进顺利。去产能、去库存、去杠杆成效明显，水泥、煤炭、钢铁分别压缩产能800万吨、299万吨、16万吨；烟花爆竹生产整体退出；中心城区房地产库存去化周期缩减为12个月，处于合理区间；政府债务管理日益规范。投融资体制改革扎实推进，市本级投融资公司现代企业制度基本建立，县域投融资公司实现全覆盖。财税体制改革稳步推进，“营改增”及各项企业减税降费政策全面落实，累计为企业减负24亿元以上。重点改革成效显著。395家市属国有企业改制圆满完成，国企国资改革持续深化。在全省率先推出商事制度“改革套餐”，“五证合一”登记制度改革全面启动。农村综合改革不断深化，金融服务“三农”在全国创造“衡阳经验”，耒阳“两权”抵押贷款和综合行政执法体制改革国家试点成效明显。城区4家信用联社成功改制组建市农商行。城市公交改革成为人民满意的民生工程。国有林场改革全面完成。不动产登记制度、公务用车制度、生态文明体制、园区管理体制等改革扎实推进。开放带动效应不断放大。全省首家综合保税区运行良好，公路口岸建成投用。深入实施“走出去”战略，累计实现外贸进出口总额102.4亿美元，总量居全省第二，对外经济合作遍及全球40余个国家和地区。累计实际利用外资38.37亿美元、内资1107.5亿元。

四是突出统筹协调发展，城乡面貌呈现新变化。城市骨架全面拉开。完成第7次城市总体规划修编。中心城区带动战略加快实施，来雁新城、滨江新区、陆家新区、酃湖高铁新城、东洲岛片区建设有序推进；累计新（扩）建衡州大道、船山路、蒸湘南路等城市主次干道22条，基本形成“三纵三横三环”的城市路网格局。交通融城战略扎实推进，县城扩容提质步伐不断加快，城镇综合承载力不断提升，全市城镇化率达50%。农村公路通畅率达100%，行政村客班车通达率达97.8%。城市功能大幅提升。南岳机场成功通航并开辟8条航线；湘桂高铁、吉衡铁路开通运行，怀邵衡铁路建设推进顺利，娄衡高速建成通车，绕城高速“三纵两横”全线贯通，铁路、高速公路总里程分别跃居全省第2、第3位；湘江土谷塘航电枢纽4台机组全部投产发电，湘江2000吨级航道衡阳至株洲段开工建设。“两供两治”建设扎实推进，全市新（扩）建污水处理厂9座，垃圾焚烧发电厂建成投产，餐厨垃圾处理中心基本建成，城市生活污水、生活垃圾处理率分别达85.7%、100%。海绵城市及地下综合管廊加快建设，“四湖一河一湿地”建设推进顺利。建成公园5个，新建“三江六岸”风光带30.8公里。获评电信普遍服务试点城市，宽带出口流量占全省35%。城市管理明显加强。统筹推进“八城同创”，成功获批全国文明城市提名城市。市区两级管理职能调整持续深化，城管、环卫、园林等职能全面下放。大力开展“城市管理年”“六大战役”“两大工程”等活动，深入实施“规电”“禁停”等专项行动，社区“两化两体系”建设和中心城区“三清三建”、主次干道两厢提质改造、农贸市场标准化改造扎实推进。烟花爆竹逐步限放禁放。殡葬改革经验在全省推介。城乡环境持续改善。圆满完成湘江保护和治理“一号重点工程”第一个“三年行动计划”，水口山、合江套等重点区域及湘江流域综合整治成效明显，省里下达的节能减排任务全面完成。县级以上饮用水水源地水质达标率稳定为100%。全面实施大气污染防治，城区空气优良率达80%。扎实开展“三边”造林、通道绿化和裸露山地造林，森林覆盖率达47%。围绕城市周边连片推进新农村建设，30个村被评为省级“美丽乡村建设示范村”或“秀美村庄示范村”。农村环境综合整治实现全覆盖，中心集镇“五乱”现象得到有效整治，农村基本不见暴露垃圾。获批全国生态文明先行示范区。

五是坚持民生为本，社会民生得到新改善。人民生活水平明显提高。坚持财政收入用于民生支出主导向，民生支出累计达1229.38亿元，占财政总支出的67.9%，年均增长17.2%。城乡居民收入稳步提高，2016年城乡居民可支配收入分别达28901元、15675元。脱贫攻坚扎实推进，全市395个贫困村实现驻村帮扶全覆盖，累计减少贫困人口25.6万，农村低保兜底脱贫经验在全省推广。农村电网第二期改造基本完成。解决205.6万农村人口饮水不安全问题。社会保障不断健全。累计新增城镇就业29万人、农村劳动力转移就业27万人。五大险种参保基本实现全覆盖，新农合参合率稳定在99%以上，企业退休人员养老金实现“十二连调”。保障性安居工程开工建设15.7万套，完成危房改造4.66万户。社会救助、社会福利体系不断完善。静州往事社会事业协调发展。全面或超额完成省、市重点民生实事98件。创建义务教育合格学校1449所，高中招生改革顺利完成，中心城区中小学“大班额”现象有效化解。公立医院综合改革有序推进，药品零差率销售实现公立医院全覆盖。生育政策调整平稳有序，出生人口性别比稳步下降。“公共文化服务进社区”荣获国家公共文化服务体系示范项目，新闻出版、广播影视、文学艺术繁荣发展。市游泳馆、体育馆建成投用，市十运会成功举办，全民健身运动蔚然成风。社会大局和谐稳定。安全生产“六大攻坚战”扎实推进。

应急管理和食品药品安全监管全面加强。平安衡阳创建深入推进，社会治安防控体系进一步健全，防范打击违法犯罪有力有效。信访工作制度改革稳步推进，房地产领域矛盾纠纷化解有力，获评2010－2015年全省信访工作先进集体。严厉打击非法集资，守住了不发生系统性和区域性金融风险的底线。民族宗教管理规范有序。

六是深入推进政府职能转变，政府自身建设取得新进展。市县政府职能转变和机构改革、并乡合村工作圆满完成。“放管服”改革不断深化，在全省率先完成权力清单动态调整，创新推行“两集中、两整合、两公开”改革，企业、政府投资项目审批时间分别提速70%、65%。市县两级“12345”政府服务热线建成投用，服务质量位列全国地级市第4位。自觉接受人大及其常委会的法律监督和工作监督、政协的民主监督以及社会各界的监督，充分采纳各民主党派、工商联和无党派人士意见、建议，累计办理市人大代表议案4件、建议1177件，办理市政协提案1448件。政府法律顾问制度积极推行。建市以来首获地方立法权，首部地方性法规《衡阳市南岳区综合管理条例》经省人大常委会批准，即将施行。“六五”“七五”普法有序衔接。第九次村委会换届选举圆满完成。党的群众路线教育实践活动、“三严三实”专题教育和“两学一做”学习教育扎实开展。坚决落实市政府党组全面从严治党主体责任，强化效能监察和审计监督，深入推进党风廉洁建设和反腐败斗争，大力建设廉洁政府，发展环境持续优化。

各位代表！四年来，我们坚决落实党在新形势下的强军目标，全力支持国防和军队现代化建设，国防动员和后备力量建设不断加强，人防工作获评全国先进，军民融合发展开创新局面。

各位代表！本届政府取得的成绩来之不易，这是省委、省政府及市委正确领导的结果，是全市人民团结拼搏、共同奋斗的结果，是社会各界人士关心支持、大力帮助的结果。在此，我代表市人民政府，向全市各族人民，向各民主党派、工商联、无党派人士、人民团体，向驻衡部队、武警官兵和政法干警，向国内外所有关心支持衡阳发展的各界朋友，表示诚挚的感谢和崇高的敬意!

回顾四年历程，我们深刻体会到：只有坚持解放思想才能赢得主动，我们正是在持续深入解放思想中不断抢抓机遇、顺势而为，才牢牢把握住了新常态下经济发展的主动权；只有坚持改革创新才能永葆活力，近年来衡阳经济社会保持平稳健康发展的良好势头，关键在于立足实际，坚定不移加快改革开放步伐、加大创新力度，让发展活力在改革创新、扩大开放中倍乘释放；只有坚持真抓实干才能奋发有为，在全市爬坡过坎的攻坚时期，我们围绕既定目标任务埋头苦干、一任接着一任干，展示了敢于担当、积极进取的责任政府形象；只有坚持执政为民才能砥砺前行，衡阳的改革发展历程告诉我们，必须把扩大群众获得感作为想问题、办事情的第一标尺，在谋求发展中集中民智、在抢抓发展中汇聚民力、在共享发展中赢得民心。我们坚信，这些宝贵的精神财富必将继续转化为推动发展的强大动力。

同时，我们也清醒认识到，我市改革发展中仍存在不少问题：经济下行压力仍然较大，发展不足、不优、不平衡的状况尚未根本改变，社会投资动力不足，企业整体创新能力不强，金融等领域存在风险隐患，财政收支矛盾比较突出，部分经济指标低于预期。新旧增长动力接续不畅，冶炼、化工、钢铁等传统支柱产业拉动作用逐步减弱，电子信息、高端装备制造等新兴产业支撑作用有待加强，城镇综合承载力不足，县域经济、工业经济、旅游经济等发展短板亟待补齐。保障改善民生任重道远，生态文明建设面临不少挑战，脱贫攻坚任务艰巨繁重，教育、医疗、住房等民生领域还存在薄弱环节，部分群众生活还比较困难。政府自身建设仍需进一步加强，“四风”问题还没有彻底根除，部分党员干部的进取精神和担当意识有待提升，为官不为、为官慢为等现象还不同程度存在，政务服务、亲商环境有待进一步改善。对此，我们必须积极应对、努力解决，确保经济发展行稳致远。

二、新一届政府工作的总体要求及奋斗目标

今后五年，是我市全面建成小康社会、开启实现基本现代化新征程的关键时期。只要我们牢牢把握目前我市所处的历史方位和阶段特征，抢抓机遇、乘势而上，就一定能在新的起点上谋求新发展、实现新突破。

今后五年，全市经济社会发展的指导思想是：高举中国特色社会主义伟大旗帜，以马克思列宁主义、毛泽东思想、邓小平理论、“三个代表”重要思想、科学发展观为指导，深入贯彻习近平总书记系列重要讲话精神，按照“五位一体”总体布局和“四个全面”战略布局，牢固树立和贯彻落实创新、协调、绿色、开放、共享发展理念，瞄准打造“现代产业强市、文化旅游名城、幸福和谐家园”，决胜全面小康，着力植优补短，坚持改革开放，全面从严治党，为建设经济繁荣、百姓富裕、生态优美、社会和谐、政治清明的新衡阳而努力奋斗。

今后五年的奋斗目标是：

——坚持植优补短，建设经济繁荣新衡阳。落实创新引领、开放崛起战略，持续厚植已有优势、全面补齐发展短板，有序推进中心城区周边县调整为市辖区，建成更具竞争力的全省第二大城市。到2021年，全市实现地区生产总值4000亿元以上，一般公共预算收入突破400亿元，固定资产投资年均增长16%以上，规模工业增加值年均增长8.5%，社会消费品零售总额年均增长12%以上。县域经济总量、一般公共预算收入均占全市的45%以上。高新技术产业产值占工业总产值的比重超过40%。旅游业真正成为富民强市的主导产业。

——坚持共建共享，建设百姓富裕新衡阳。2018年率先实现整体脱贫，2020年如期全面建成小康社会。到2021年，全体居民人均可支配收入年均增长9%以上，城镇登记失业率控制在4.5%以内；每千人医疗床位数达6.0张，社区医养结合日间照料服务覆盖率达30%，基本医疗保险覆盖率超过95%，建成区域性医疗卫生中心。建立文化旅游融合发展体系，成功申报国家历史文化名城，推进南岳衡山申遗工作。加快构建综合交通枢纽体系。市民文明素质与受教育程度明显提高。成功创建全国文明城市。

——坚持绿色发展，建设生态优美新衡阳。生态保护与环境治理全面加强，能源资源开发利用效率大幅提高。大气环境质量、重点流域和重要水库水质达标率、饮用水安全保障水平稳步提升，湘江干流及主要支流水质稳定在Ш类标准以上，全面完成节能减排目标任务。加快建设全国生态文明先行示范区。到2021年，全市城镇化率达56.2%，森林覆盖率超过50%，空气优良率超过85%，县级以上污水处理率达95%，生活垃圾资源化利用率达35%，90%的村庄生活垃圾得到有效处理。

——坚持以人为本，建设社会和谐新衡阳。始终把维护人民合法权益摆在首要位置，扎实办好省、市重点民生实事。基层群众自治组织和社会组织发展壮大，多方参与的现代社会治理体系不断完善。公共突发事件防范和处置、防灾减灾能力大幅增强。信访法治化改革深入推进，社会大局持续和谐稳定。

——坚持依法行政，建设政治清明新衡阳。中国梦和社会主义核心价值观更加深入人心。社会主义民主政治全面加强，法治政府基本建成。司法透明度和公信力明显提高，人民权益和社会公平正义得到切实保障。全面从严治党深入推进，干部作风持续好转，腐败问题得到有效遏制，政治生态风清气正。

三、2017年的工作安排

2017年是新一届政府的开局之年，是实施“十三五”规划的关键之年，是供给侧结构性改革的深化之年。做好2017年政府各项工作，必须全面落实中央、省委、市委经济工作会议精神，坚持稳中求进工作总基调，以推进供给侧结构性改革为主线，全力做好稳增长、促改革、调结构、惠民生、防风险各项工作，努力实现经济社会持续健康发展。

2017年全市经济社会发展主要预期目标是：地区生产总值增长8.5%左右，一般公共预算收入增长7%左右，规模工业增加值增长7.5%左右，固定资产投资增长14%左右，社会消费品零售总额增长12%左右，全体居民人均可支配收入增长8.5%左右，全面完成省里下达的节能减排、安全生产等约束性指标。

围绕以上目标，重点做好以下工作：

（一）着力推进植优补短，确保经济平稳健康发展

加快县域经济发展。发展特色产业。引导和支持各县（市）结合自身优势和产业基础，分别打造1-2个具有鲜明特色的主导产业：衡南重点发展高端装备制造、空港物流产业，衡阳县做大做强生物医药、陶瓷产业，衡山提质发展旅游、汽车零部件制造产业，衡东加快发展机械制造、化工产业，祁东重点推进新材料、农副产品深加工产业，常宁致力打造全省最大的水口山铜铅锌深加工综合基地、油茶生产基地，耒阳着力发展工矿业、煤电冶炼产业。抓实重点项目。积极实施重大项目带动战略，大力推进五矿金铜（二期）、特变电工南方智能电网科技产业园等项目建设，重点支持中科光电、恒生制药等骨干民营企业发展壮大。挖掘园区潜力。各县（市）按照“先建园区再引项目”的思路，加快园区规划编制及基础设施建设，加大重大产业项目招引力度，深化园区管理体制改革，着力打造县域经济增长极。

加快工业经济发展。提升制造业层次和水平。实施制造强市“663行动计划”，打造全省先进制造业基地。加快产业集聚集群发展。着力发挥细分行业领军企业的辐射带动作用，支持富士康南方精密模具生产基地、中兴衡阳科技园、镭目科技核辐射安全监测成套设备研发及产业化发展，加速形成电子信息产业集群；支持特变电工智能电网配套、湖南机油泵节能汽车零部件智能制造、天雁机械涡轮增压器、南岳电控高压共轨项目建设，加速形成高端装备制造产业集群；支持五矿金铜、建滔化工、春昌有色等企业延长产业链，加速形成有色金属加工产业集群。支持种子期、初创期成长型中小企业发展，推动小微企业转型升级为规模企业。提质升级市直工业园区。优化“一核一圈二带”工业产业空间布局，推进产城融合发展，加快衡山科学城基础设施建设和高新区、白沙洲工业园、松木经开区扩区提质。

加快旅游经济发展。统筹规划“一龙头、一中心、两圈、三带、四区”空间布局，加快旅游产品开发，打造全域旅游示范区。启动南岳衡山旅游经济新区建设，抓好方广寺禅修文化养生基地、传奇古镇等重点项目建设。打破封闭性景点景区建设和经营模式，支持各县（市）对接南岳打造精品旅游景区和线路，加快庙前、蔡伦竹海、岣嵝峰、四明山、岐山、四方山等景区提质改造。积极申报雨母省级风景名胜区，建设城区后花园。抓好酃湖公园、青草桥风情休闲街等景区建设，支持石鼓书院、东洲岛、雁峰打捆创建5A级景区。大力发展乡村旅游，推进宝盖、渣江、归阳、新市等古镇的保护和开发，积极创建全国休闲农业与乡村旅游示范县、示范点。探索推进旅游市场和景区管理体制改革，加强风景名胜区保护，强化基础设施配套，实施“旅游服务质量提升行动”和“引客入衡”工程，力争全市接待国内外游客、实现旅游总收入分别增长15%、24%。

（二）全面深化改革开放，不断增强经济发展的动力活力

坚决落实创新引领战略。加强创新平台建设，加快构建区域性科技创新中心，建立多层次工程技术研究中心、重点实验室和企业技术中心，推进衡山科学城科技创新中心、高新区双创中心等一批科技孵化器和众创空间建设，力争高新技术企业突破100家。鼓励企业加大研发投入，重点突破核级海绵锆（铪）、重型共轨供油泵、国六喷油器等关键技术。推动产学研合作体制机制创新，支持园区、企业与高等院校、科研机构等加强科技信息对接，合作开展核心技术、共性技术、关键技术研发与攻关，加快创新成果转移转化。启动企业与在衡高校联合培养人才计划，加强专业人才培养和智力引进，加快建设人才强市。

深入推进供给侧结构性改革。继续推动钢铁、煤炭、水泥等行业化解过剩产能，防止已化解的过剩产能死灰复燃，有序处置“僵尸企业”。支持合理自住购房，加快发展机构化、规模化住房租赁企业，科学合理供应住宅用地，促进房地产市场平稳健康发展。完善支持金融发展的政策体系，建立政银企对接长效机制，加大股权融资力度，降低企业杠杆率。下决心处置一批风险点，着力防控资产泡沫。大力振兴实体经济，提高金融支持实体经济的精准度和实效性，加大减税、降费、降低要素成本工作力度，推动企业眼睛向内降本增效。引导企业发扬“工匠精神”，加强品牌建设，培育“百年老店”。

着力促进关键领域改革落地。深化国有企业改革，健全国有资产监管体制，推进混合所有制改革，加快驻衡中央、省属企业“三供一业”分离移交。加强投融资体制改革，努力降低融资成本，大力推广PPP模式。深入推进公共资源交易体制改革，实现阳光交易。深化商事制度改革，加强事中事后监管。大力推进农村综合改革，重点推动农村土地“三权分置”，抓好农村产权制度和新一轮农垦体制改革。有序推进户籍制度改革，全面实施居住证制度，加快农业转移人口市民化。统筹推进医药卫生、财税、价格、生态文明等体制改革。

全面提升开放型经济发展水平。坚持开放崛起战略，提升开放平台功能，加快建设开放强市，打造更具辐射力的区域性中心城市。支持有色、钢管等优势产业抱团出海，转移优质剩余产能，进出口总额增长8.5%左右。重点围绕龙头企业开展建链补链强链招商行动，着力引进一批行业领军企业，努力提升招引的质量效益。做好外事侨务和港澳台工作，充分发挥异地商会作用，引进有实力的外地客商来衡投资兴业。

（三）统筹推进“三农”发展，加快农业现代化步伐

发展现代农业。大力实施新增粮食产能工程，确保粮食总产量稳定在330万吨以上。全面推进“十百千万”现代农业发展工程，支持大三湘、绿海粮油、角山米业等一批农产品加工企业做大做强，培育领军型农业产业化龙头企业10家以上，力争全市农产品加工业总产值增长20%以上。加快国家现代农业科技示范园、九龙生态等现代农业特色产业园建设，促进一二三产业融合发展。着力打造以精细农业为特色的优质农副产品供应基地，加快发展蔬果、油料、茶叶、烟叶等高效经济作物，支持油茶加工主导行业标准制定。实施新型职业农民培育工程，推进多种形式的适度规模经营，构建新型农业经营体系。强化农业科技创新与推广应用，加大种质资源创新和新品种开发力度，不断提高农业机械化水平。加强“五小”水利工程建设，着力解决农田灌溉“最后一公里”问题。

建设美丽乡村。开展新农村建设提质升级行动，重点打造雨母山、茶山坳、横江、萱洲等一批高档次、有品位的美丽乡村特色片区，常态化推进农村环境综合整治，力争全市文明村镇达标率在30%以上。加快完善农村公路、农田水利、自来水、网络宽带等基础设施。规范农村新建、扩建、改建房屋，鼓励引导村民到农村集中居住点建房，大力推进农村“空心房”拆除工作。

加快脱贫攻坚。全面落实十大扶贫举措，按照“四跟四走”思路，加大产业扶贫力度，扎实推进易地扶贫搬迁和农村危房改造。加大扶贫小额信贷支持力度。加强农村最低生活保障制度与扶贫开发政策的有效衔接，将符合政策规定的贫困人口全部纳入农村低保范围。确保贫困村全部通电、通路、通电视、通宽带。力争贫困村居民人均可支配收入增长10%以上，贫困县“摘帽”、贫困村退出、贫困人口基本脱贫。

（四）推进服务业集聚发展，着力打造区域性服务中心

全面放宽服务业市场准入。推进服务业市场便利化改革，进一步放开银行、证券、保险、电信、邮政快递等行业市场准入，鼓励社会资本进入教育、医疗、健康、文化等领域，加快形成统一开放、公平竞争的市场体系。积极扩大向社会购买基本公共服务的范围和比重。规范完善政府采购办法，加快公共资源配置市场化。

引导传统服务业创新发展。繁荣商贸服务业，提质改造老旧商业区，加快华耀城、万达广场、丽波酒店、衡阳国际会展中心建设，规划建设、培育壮大新兴专业市场，推动批发零售、住宿餐饮、休闲娱乐等生活性服务业向精细化和高品质转变。完善市场体系，建设华新跨境电商产业园，加快推进乡镇配送中心和农村电子商务网络建设。

推动现代服务业加速发展。做强现代物流业。努力建设全国重要物流枢纽城市。发挥临铁、临空、临港优势，发展云集空港物流产业，加快大浦通用机场建设；加快白沙洲物流园、衡缘物流园及唯品会配送、衡东仓储物流中心建设，支持品牌快递企业在衡建立区域转运中心。壮大金融服务业。加快金融信用信息服务中心建设与运用，健全金融信用信息服务体系；吸引各类金融机构来衡设立分支机构，实现全市县域村镇银行全覆盖。大力发展总部经济、科技研发、文化创意、汽车后市场等业态。

（五）加快构建新型城镇体系，建设安居乐业美好家园

增强中心城区辐射能力。实施第8次城市总体规划修编，推进中心城区区划调整，加强重要地段、街区的城市设计。启动城市轨道交通前期工作，加快推进怀邵衡铁路衡阳段、瓦松铁路及湘江2000吨级航道衡阳至株洲段等项目建设，加快二环路、北三环形象进度，基本完成中心城区主次干道提质改造。深入推进交通融城战略，加快衡州大道西延、衡岳大道北延、东风路南延建设。加大来雁新城、滨江新区、陆家新区、酃湖高铁新城、东洲岛片区建设开发力度。推进蒸水北堤、湘江西岸风光带建设。大力建设海绵城市、智慧城市，构建城市防洪防涝、山洪地质灾害防御体系。加强城市供水及能源通道建设。加快旧城改造步伐，加大政府购买棚改服务力度。

实施县城建设攻坚行动。支持各县城对标中心城区强化基础设施配套，不断完善服务功能，努力提高管理水平，真正让县城成为各县（市）推进新型城镇化的主阵地。衡南全力推进新县城“两带四新区”建设开发，衡阳县加快西渡高新区核心片区建设和船山西路沿线开发，衡东、衡山与南岳区融合打造南岳-开云-新塘组团，祁东着力推进撤县设市，常宁有序实施“三年兴城行动”，耒阳加快打造中等规模城市。

差异化推进小城镇建设。推进西渡、樟木、云集、三塘、车江、店门、南岳等城镇与中心城区高效联动、一体发展。各县（市）每年抓好1-2个特色集镇建设，打造一批经济强镇、特色小镇。提高市、县边际中心城镇建设水平。完善城镇水、电、路、讯等基础设施和环卫、绿化等配套设施，启动新一轮农村电网改造升级，加快国省干线公路和农村公路提质改造步伐，大力开展路域环境和超限超载整治，推进农村信息基础设施三年行动计划。

提高城市管理水平。深入开展全国文明城市创建工作，充分运用“数字衡阳”建设成果，推进信息采集服务外包。扎实推进城市执法体制和管护市场化改革，强化交通动态管控和智能化管理。加强城市停车设施建设管理。严格控违拆违，加大建成区违法建设清理整治力度。深化社区“两化两体系”建设，全面完成中心城区“三清三建”和农贸市场标准化改造，加快推进人行天桥和地下通道建设，深入开展占道经营、乱停乱放、渣土污染和火车站、高铁站周边环境等专项治理，全面推进城区烟花爆竹禁放限售工作，让城市更洁净、更美丽、更有序。

（六）致力改善生态环境，努力建设美丽衡阳

推进低碳发展。建立健全绿色发展引导机制和政策体系，积极参与碳排放交易市场建设。大力推进“气化衡阳”工程，加快煤改气、电改气、油改气步伐。鼓励绿色出行，加强公共自行车租赁系统建设，提高城市公共交通运输比重，扩大新能源交通工具使用范围，推动城区公共交通加速向“零排放时代”迈进。加快淘汰改造10蒸吨以下燃煤锅炉，全面完成黄标车淘汰任务。推广装配式建筑和绿色建材，加快住宅产业化步伐，确保政府投资的公建项目产业化建设全覆盖。

抓好污染防治。扎实推进湘江保护和治理“一号重点工程”第二个“三年行动计划”，强化水口山、松江、大浦、合江套等重点区域污染治理，全面封堵蒸水两岸排污口，巩固提升规模养殖污染防治成果，确保湘江、蒸水城区段水质稳定在Ⅲ类标准以上。加快雁栖湖等城市黑臭水体整治。加强河道采砂、船舶、码头污染执法监管和饮用水水源保护，确保县级以上饮用水水源水质达标率稳定为100%。推行污染源在线监测设施全覆盖试点，完成角山、松木、酃湖等污水处理厂建设，中心城区、主要工业园区基本实现污水全收集、全处理；加快重点建制镇污水处理设施建设。推进城镇垃圾转运站、垃圾填埋场建设。

强化生态保护。努力争创国家园林城市、国家环境保护模范城市。严守环境质量底线、耕地保护和生态保护红线。加快推进横江、幸福河等湿地公园建设。全面推行河长制，加强河湖空间用途管制，维护河湖健康生命。健全生态保护补偿机制，加快山水林田湖生态修复及重点土壤污染治理修复项目建设。加大祁东、衡南国家水土保持重点治理工程实施力度。强化人工造林、封山育林和森林管护，巩固提升“三边”造林、通道绿化、村庄绿化和砂场复绿成果。力争全市森林覆盖率达47.6%、城市建成区绿化覆盖率超过42%。

（七）积极弘扬先进文化，着力打造文化名城

丰富群众精神文化生活。深入开展中国梦和社会主义核心价值观教育。大力推进公民思想道德建设工程，加强未成年人思想建设和大学生思想政治工作，培育传承良好家风家教、校风校训、企业精神和乡贤文化。组织开展群众性精神文明创建活动，不断提升社会文明程度。继续实施文化惠民工程，提升“周末百姓剧场”等群众文化品牌影响力，促进基本公共文化服务标准化、均等化。加快市影剧艺术中心、市图书馆、市艺术学校实验剧场等项目建设。

加快国家历史文化名城建设。大力挖掘并弘扬具有衡阳个性特征的优秀传统文化。加强名人名居及纪念设施保护展示，加大船山书院、申公馆、来雁塔、珠晖塔、接龙塔、湘江东路等文物古迹、历史建筑、历史街区的保护和修缮力度。加强张家山抗战遗址、酃县遗址、云集古窑遗址的保护与开发。

增强文化产业竞争力。挖掘整理并传承保护好衡州花鼓戏、衡阳湘剧、祁剧、衡山影子戏、南岳庙会等地方传统文化，努力申报一批国家、省级非物质文化遗产。提升壮大传统文化产业，鼓励发展新型文化业态，推进文化与科技融合发展，培育和引进一批有实力的文化企业。加快衡阳文化“走出去”步伐，讲好衡阳故事，传播衡阳声音，扩大衡阳影响。

（八）促进社会事业全面发展，切实保障改善民生

做好就业托底工作。加强创新创业孵化基地建设，做好“双百资助工程”、省级示范基地、“2151工程”申报推荐工作。实施“雁归兴衡”行动计划，加大大学生自主创业奖补扶持力度，支持大学生创业园区建设。积极开展职业技能培训和创业培训，做好高校毕业生、就业困难人员、农村转移劳动力、退役军人等重点群体的就业工作，确保城镇登记失业率控制在4.5%以内。

打造人民满意教育。落实教育优先发展战略，全面提升教育教学质量。完成义务教育学校标准化建设试点，规范、扶持发展义务教育阶段非营利性民办教育，推进县域内城乡义务教育一体化建设。扩大学位供给，努力解决“大班额”“择校热”和进城务工人员子女上学难等问题。加快市铁一中、市九中搬迁，逐步普及高中阶段教育。推进市中小学素质教育综合实践基地、职业教育产教融合示范园区建设。继续实施第二轮学前教育三年行动计划。支持在衡高校发展，加快南华大学新校区建设。大力发展继续教育。办好特殊教育。强化教师队伍建设，推进教师职称制度改革，深入实施“名师工程”，逐步提高教师待遇。加强学校周边环境综合治理。

建设健康衡阳。加强公共卫生服务，推进医疗卫生信息平台和分级诊疗制度建设，建立覆盖城乡的基本医疗卫生制度。强化重大疾病疫情防控。大力发展健康产业，加强社区医养结合、居家养老日间照料等服务能力建设。加强重点专科建设，支持中医药事业发展。完善医疗纠纷调解机制，依法严惩涉医犯罪和“医闹”行为。坚持计划生育基本国策，促进人口均衡发展。广泛开展全民健身运动，扎实做好省十三运会和省第十届残运会筹备工作，加快市体校搬迁，促进群众体育和竞技体育全面发展。

完善社会保障体系。实施全民参保登记计划，健全参保缴费激励约束机制，提高统筹层次。鼓励发展政策性保险及商业保险。发展社会福利和慈善事业，完善临时救助和重大疾病医疗救助制度，积极关爱留守儿童和孤寡老人。全面完成保障性安居工程建设任务。加强国有土地上房屋征收和集体土地上房屋拆迁货币结算安置工作。

加强社会治理创新。坚持安全发展，落实安全责任，强化重点行业和领域安全监管，完善自然灾害、突发事件监测预警和应急处理机制，全面加强食品药品安全监管体系建设。扎实推进社会治理“四化一制”，加快“雪亮工程”等治安防控体系建设，推行网格化管理，加强禁毒工作，严厉防范、依法惩处各类违法犯罪行为。加强和改进信访工作，完善矛盾纠纷多元化解机制。把防控金融风险放到更加重要的位置，规范民间融资，加快构建地方金融组织的过程监管体系，确保不发生系统性和区域性金融风险。依法管理民族宗教事务和场所，深入开展民族团结进步创建“六进”工作。积极创建社会管理综合治理优秀城市。

（九）加强政府自身建设，提高行政能力和服务水平

坚持依法行政，加快法治政府建设。深入开展“七五”普法宣传。健全重大决策公众参与、专家论证、合法性审查、集体决定、跟踪评估和责任追究机制。党政机关普遍建立法律顾问制度，加强和改进行政应诉工作。不断完善政府合同管理。积极推动雨母山生态保护立法。深化行政执法体制改革，推行行政执法公示、执法全过程记录、重大执法决定法制审核三项制度试点工作，探索委托乡镇开展行政执法。自觉接受人大及其常委会的法律监督和工作监督、政协的民主监督以及社会各界的监督，认真办理各级人大代表议案建议、批评、意见和政协提案。支持监察、审计、法制等部门依法开展工作。

坚持科学施政，提升服务发展水平。加快简政放权，深化“两集中、两整合、两公开”改革，深入推进“互联网+政务服务”，让群众和企业少跑腿、好办事。全面推进行业协会商会与行政机关脱钩，建立完善综合监管体系。构建“亲”“清”新型政商关系。加强并乡合村后续管理，加快农村基层综合服务管理平台建设，推进便民服务向基层全覆盖。健全有利于科学发展的考评机制、容错纠错机制及绩效管理体系，完善政府债务管理和风险防控机制。

坚持从严治政，切实改进工作作风。继续扩大财政预决算公开范围，严格控制“三公”经费支出。深化和扩大政务公开，全面公开“三清单一目录”及政府权力运行流程。严格实行首接首办制，重点整治行政不作为、乱作为、慢作为等问题，坚决纠正推诿扯皮、效率低下等行为，确保政令畅通、执行高效。完善提升“12345”政府服务热线功能。

坚持廉洁从政，提升拒腐防变能力。全面推进惩治和预防腐败体系建设，建立健全廉洁风险防控体系。严格执行领导干部重大事项报告制度。加强对国家公职人员行使权力的监督，严禁领导干部插手政府采购、工程招标、土地矿产拍卖等经济活动。坚决整治和查处侵害群众利益的不正之风和腐败问题，努力建设廉洁政府。

加强国防和后备力量建设。支持国防和军队改革，广泛开展全民国防教育、国防动员和“双拥”共建活动，争创全国双拥模范城市。做好人民防空和军事设施保护工作。推进军民技术双向转移和融合发展，扎实推进白沙绿岛军民融合产业园建设，开创军政军民团结新局面。

各位代表！立足新起点，展望新征程，我们深感责任重大，使命光荣。让我们紧密团结在以习近平同志为核心的党中央周围，在市委的坚强领导下，团结和带领全市人民，凝聚共识、锐意进取、开拓创新，为建设“五个新衡阳”、加快全面建成小康社会而努力奋斗！
